# Dose-response and isotemporal substitution analysis of domain-specific physical activity and sedentary behavior with abdominal aortic calcification risk: A cross-sectional study

**DOI:** 10.1371/journal.pone.0332964

**Published:** 2025-10-07

**Authors:** Zhuohui Yang, Lin Zhu, Zekai Chen

**Affiliations:** 1 School of Exercise and Health, Guangzhou Sport University, Guangzhou, China; 2 Guangdong Provincial Key Laboratory of Physical Activity and Health Promotion, Guangzhou, China; 3 Key Laboratory for Exercise and Adolescent Physical Health, Guangzhou Sport University, Guangzhou, China; Saga University, JAPAN

## Abstract

**Background:**

Abdominal aortic calcification (AAC) is an independent risk factor for cardiovascular disease. This study aims to examine the dose-response relationships between domain-specific moderate-to-vigorous physical activity (MVPA), sedentary behavior (SB), and AAC risk, and the effects of time reallocation among these behaviors, in individuals aged 40 years and older.

**Methods:**

This cross-sectional study used data from NHANES participants from the 2013–2014 cycle. MVPA and SB were self-reported, and AAC status was assessed using the Kauppila scoring system and DXA. Weighted logistic regression calculated odds ratio (OR) and 95% confidence interval (CI) for dose-response relationships, using restricted cubic splines (RCS). Furthermore, weighted logistic regression models within an isotemporal substitution analysis were employed to examine the effect on AAC risk of reallocating time among different MVPA domains and sedentary behavior.

**Results:**

This study included 2,842 participants (median age 58 years, interquartile range [IQR] 48−68 years, 48.42% male), of whom 861 (30.30%) had AAC. Adherence to physical activity (PA) guidelines (≥150 minutes/week) for leisure-time MVPA was associated with a 33.7% reduction in AAC risk (OR = 0.643, 95% CI 0.488–0.848, P = 0.035). However, no significant effect of occupation and transportation-related MVPA on reducing AAC risk was found. RCS revealed dose-response relationships between total MVPA, leisure-time MVPA, and SB with AAC risk, indicating a U-shaped pattern for total MVPA, with the lowest risk at 1086 minutes/week (OR = 0.712, 95% CI 0.546–0.928, non-linear P = 0.023). Moreover, isotemporal substitution analysis showed that replacing 30 minutes/day of sedentary behavior (OR: 0.837, 95% CI: 0.747–0.927) or occupational MVPA (OR: 0.842, 95% CI: 0.692–0.992) with leisure-time MVPA was significantly associated with lower AAC risk.

**Conclusion:**

There is a positive linear dose-response association between sedentary behavior and AAC risk; conversely, leisure-time MVPA shows a negative linear dose-response association. Total MVPA presents a nonlinear dose-response association, with AAC risk being lowest when activity reaches 1086 minutes per week. Isotemporal substitution analysis further revealed that reallocating time from sedentary behavior or occupational MVPA to leisure-time MVPA is associated with a lower risk of AAC. These results suggest that increasing leisure-time MVPA and reducing sedentary behavior may help optimize AAC risk.

## Introduction

Cardiovascular diseases (CVDs) are a major contributor to the global burden of disease, leading to substantial health loss and significant costs to healthcare systems [1]. For individuals aged over 50, the leading causes of death were ischemic heart disease and stroke, respectively [2]. The American Heart Association reported that the crude incidence of CVD in 2020 increased by 29.01% compared to 2010 [3]. Cardiovascular health was influenced by multiple factors, with lack of physical activity and increased sedentary behavior (SB) being major independent predictors of cardiovascular health problems and preventable risk factors for many chronic diseases, including CVD [4].

Vascular calcification (VC) refers to the atypical accumulation of calcium and phosphate within the walls of arteries [5], and is identified as a contributing factor to cardiovascular diseases [6]. AAC and coronary artery calcification (CAC) are prevalent types of VC [7]. Several studies [8–11] confirmed that AAC predicted cardiovascular diseases and all-cause mortality. Recent studies [12] also showed that CAC was a powerful tool for predicting the risk of atherosclerotic cardiovascular disease. However, AAC was proven to be an equivalent or superior predictor of cardiovascular diseases and mortality compared to the Framingham risk score and CAC [10,13,14].

It has been found that there is a dose-response relationship between physical activity and the reduction of all-cause mortality and cardiovascular event risk [15,16]. In the United Kingdom’s Whitehall II cohort study [17], physical activity was unrelated to CAC in older adults. Another study [18] suggested that physical activity might protect against all-cause mortality in patients with higher CAC scores. Conversely, recent research [19] indicated that physical activity was positively associated with the prevalence and progression of CAC. These inconsistent findings indicate that the link between physical activity and vascular calcification risk remains uncertain. The 2018 Physical Activity Guidelines for Americans (2nd edition) outlined the types and durations of physical activity that significantly improve health. Guidelines suggest adults aim for 150–300 minutes of moderate or 75–150 minutes of vigorous physical activity weekly, or a mix of both, to acquire significant health benefits [20]. A recent prospective cohort study [21] confirmed that in low and middle-income countries, prolonged sedentary time is associated with an increase in all-cause mortality and CVD risk. Reducing sedentary time and increasing physical activity may be crucial strategies for alleviating the global burden of premature death and cardiovascular diseases. In 2020, the World Health Organization updated its guidelines, advising for the first time to reduce sedentary time and replace it with physical activity [22]. These findings suggest that a lifestyle incorporating more physical activity and less sedentary behavior offers significant health benefits.

Prior research by Sheng et al. [23] has investigated the association between physical activity, sedentary time and AAC. Building upon this foundation, our current study utilizes the identical cohort of participants, employing the same questionnaire to assess specific domains of physical activity, sedentary time and focusing on the same outcome, abdominal aortic calcification. Crucially, while Sheng et al. primarily examined the retarding effects of physical activity and reducing sedentary time on AAC severity after its onset, our research addresses distinct yet complementary questions related to the risk of developing AAC. Our study specifically addresses a critical gap in understanding the complex relationship between physical activity, sedentary behavior, and the risk of AAC. First, we examine the dose-response association between various domains of physical activity and sedentary time in relation to AAC onset. This is important because relying on linear assumptions may oversimplify the true relationship, potentially leading to inaccurate public health recommendations. By investigating the dose-response relationship, we aim to identify the optimal level of physical activity for preventing AAC. Secondly, we utilize the isotemporal substitution model to examine the potential impact of reallocating time among different behavioral activities. This methodological framework enables us to quantify how shifting time from one activity to another influences health outcomes, thereby providing insights that can inform personalized intervention strategies and targeted public health policies aimed at mitigating AAC risk. While acknowledging the importance of examining various physical activity intensities, our analysis concentrates primarily on MVPA. This focus is justified by MVPA’s endorsement as a standardized, widely recognized indicator aligned with contemporary public health guidelines, facilitating comparability and applicability of our findings.

To date, studies [23–25] on PA and AAC have primarily focused on slowing the progression of AAC. Few studies have explored the connections between AAC risk and three distinct domains of physical activity. Since physical activities in different domains have varying impacts on cardiovascular health [26,27], and these differences may be influenced not only by the type and intensity of the activity itself but also by unique environmental exposures and induced physiological and pathological responses within each domain, clarifying the relationships between domain-specific physical activities and the risk of AAC is essential. For instance, the potential benefits of transport-related physical activity may be attenuated by exposure to traffic pollution [37,38]. Similarly, certain types of occupational physical activity, particularly those associated with stress or high physical load, have been linked to less favorable physiological responses [40–42], which are key drivers of vascular calcification [43,44]. Consequently, understanding the relationship between physical activities specific to different domains and the risk of AAC is crucial.

This study utilizes NHANES data to explore the associations between physical activity across different domains, sedentary behavior, and the risk of AAC, including dose-response relationships and the effects of time reallocation among these behaviors.

## Methods

### Data sources

This study utilizes data from NHANES, a representative cross-sectional survey that collects health and nutrition information from the U.S. population. Data from participants aged 40 and older during the 2013–2014 cycle are analyzed, as AAC scoring was only conducted for this age group. All study participants gave their informed agreement, and the National Center for Health Statistics’ (NCHS) Institutional Review Board granted approval for the research.

**Fig 1:**
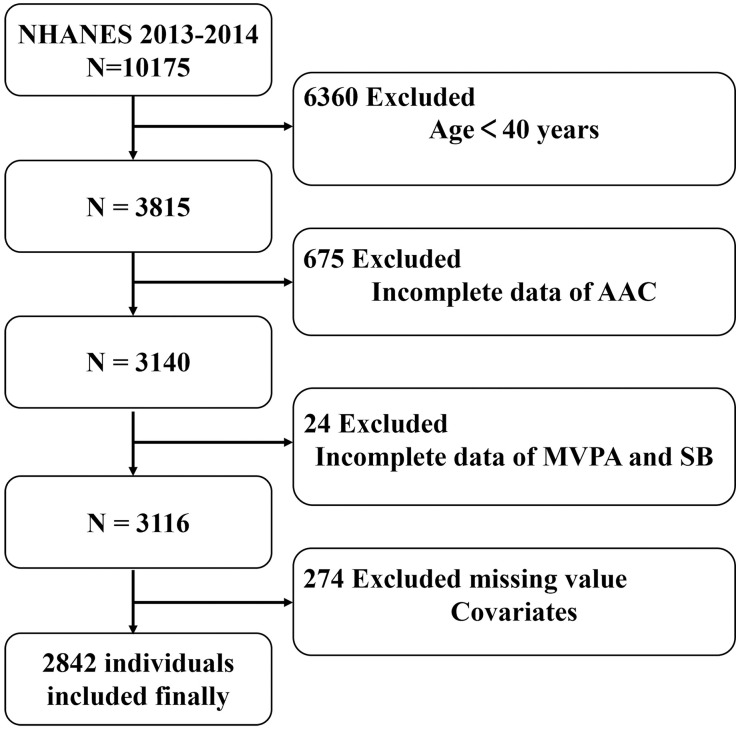
Participant Flow Diagram for NHANES 2013–2014 Sample Selection. Abbreviation: NHANES, National Health and Nutrition Examination Survey; AAC, abdominal aortic calcification; MVPA, moderate-to-vigorous physical activity; SB, sedentary behavior.

The study involved participants who had completed AAC measurements, self-reported data on moderate-to-vigorous physical activity (MVPA) and SB, and relevant sociodemographic variables. A total of 274 individuals were excluded due to incomplete data on covariates, including poverty-to-income ratio (PIR, N = 249), body mass index (BMI, N = 21), educational level (N = 2), smoking status (N = 1), and marital status (N = 1). The final analysis includes 2842 participants. Fig 1. presents a comprehensive flowchart illustrating the process of participant selection.

### Assessment of MVPA and SB

All participants filled out the Global Physical Activity Questionnaire (GPAQ), a validated measure for gathering information on physical activity and sedentary behavior across many domains [28]. The GPAQ comprises 15 questions designed to evaluate the intensity (moderate and vigorous), frequency (days per week), and duration (minutes) of physical activities across three domains: occupation, transportation, and leisure-time. Additionally, one question addresses sedentary behavior. Data on weekly MVPA in the domains of occupation, transportation, leisure-time, and daily sedentary behavior time are collected.

The calculation of MVPA involved adding the minutes of moderate-intensity activity per week to twice the minutes of vigorous-intensity activity [29]. Total MVPA time was obtained by summing the reported MVPA times across all domains [29]. Based on the U.S. Physical Activity Guidelines [20], participants were classified as “inactive” (not meeting guidelines) or “active” (meeting guidelines), with “inactive” defined as engaging in less than 150 minutes per week and “active” as 150 minutes or more per week. Recent studies [30] suggested converting daily sedentary minutes to hours and categorizing them into four groups (0 to <4, 4 to <6, 6–8, and ≥8 hours).

### Abdominal aortic calcifcation

The severity of AAC was evaluated by utilizing the Kauppila grading method to analyze lateral spine images derived from dual-energy X-ray absorptiometry (DXA). Participants excluded from the DXA examinations included those under 40 years old, pregnant women, individuals who had recently used radiographic contrast agents, those weighing over 450 pounds, and individuals with Harrington rod-associated scoliosis.

The AAC-24 score was determined by assessing the calcification of the aorta’s front and back walls in sections aligned with the lumbar vertebrae L1 to L4. A total score between 0 and 24 was obtained by rating each section on a scale from 0 to 3. The study included two groups: the no AAC group (total AAC score = 0) and the AAC group (total AAC score > 0, indicating the presence of AAC) [8,31]. Participants with an AAC-24 score of ≥1 have AAC [32].

### Demographic characteristics and other covariate

Key sociodemographic variables assessed included age, gender (male or female), race/ethnicity (Mexican American, other Hispanic, non-Hispanic White, non-Hispanic Black, non-Hispanic Asian, and other races), BMI, marital status (married/living with a partner or single), education level (high school or less, some college or associate degree, college graduate or higher), and PIR. The PIR, reflecting the relationship between income and family needs, was calculated based on family income relative to the federal poverty threshold [33]. PIR ranged from 0 (no income) to 5 (five times or above the federal poverty level). BMI is determined by dividing an individual’s weight in kilograms by the square of their height in meters. Participants’ educational levels and marital status were defined according to previous research [34].

Hypertension was characterized by self-reported use of antihypertensive medication, a doctor’s diagnosis of hypertension, Systolic blood pressure (SBP) of 140 mmHg or higher, or Diastolic blood pressure (DBP) of 90 mmHg or higher. SBP and DBP measurements were taken for individuals not on antihypertensive medication [35]. The average SBP and DBP were calculated according to CDC guidelines [36]. Additionally, DBP readings of zero were not included in the average calculation. The diagnostic criteria for diabetes were consistent with previous studies [34].

### Statistical analysis

The Shapiro-Wilk test checks if data follow a normal distribution. Categorical variables are presented as n (percentage), and continuous variables as mean (standard deviation) or median (IQR), depending on their distribution. Non-normally distributed continuous variables are compared with the Wilcoxon rank-sum test, while customarily distributed data are analyzed with the independent sample T-test. Categorical variables are examined using the chi-square test to assess the relationships between total MVPA, sedentary time, different domains of MVPA, and AAC prevalence, with Bonferroni correction applied for post-hoc comparisons.

Weighted univariate and multivariate logistic regression investigate the associations between MVPA, sedentary behavior, and AAC risk. Results are reported as odds ratio (OR) with 95% confidence interval (95% CI). Model 1 adjusts for age and sex. Model 2 includes adjustments for race, marital status, BMI, PIR, and educational level. Model 3 further adjusts for smoking status, hypertension, and diabetes. Based on Model 3, restricted cubic splines (RCS) were used to investigate the dose-response relationship between MVPA, sedentary behavior, and AAC risk and examine potential nonlinear associations. Three knots were placed at the 10th, 50th, and 90th percentiles.

Furthermore, We use the isotemporal substitution analysis [37], which examines how reallocating time among different activity types affects AAC risk, assuming total behavior time remains constant. Total behavior time is defined as the sum of all measured activities, including various domains of MVPA and sedentary behavior. In the isotemporal substitution models, we include all relevant behavior categories, designating one as the reference category, which is omitted from the model. This setup allows us to analyze the effects of reallocating time from the reference category to other behaviors while controlling for potential confounders through relevant covariates. Results are presented as odds ratios (OR) with 95% confidence intervals (CI). Each OR indicates the estimated change in the odds of AAC associated with substituting 30 minutes from the reference category with one unit of the corresponding included behavior. A statistically significant association is indicated if the 95% CI for the OR does not include 1.

All data analyses and RCS plotting are performed using R language (version 4.3.2) with Rstudio software. Weighted analyses are conducted using the Mobile Examination Center (MEC) weights provided by the R (version 4.3.2) “survey” package, accounting for the complex, multi-stage (stratified and clustered) sampling design of NHANES. Statistical significance is defined as having a P value less than 0.05 for all analyses.

## Results

### Participant characteristics

After implementing the inclusion and exclusion criteria, the study comprised 2842 eligible participants (Fig 1). All participants were categorized into two groups: the group without AAC (AAC = 0) and the group with AAC (AAC > 0). Baseline characteristics were presented in S1 Table. All participants’ median age (IQR) was 58.0 years (48.0–68.0); 48.42% were male and 51.58% were female. Among them, 861 (30.30%) were diagnosed with AAC, and 1981 (69.70%) were diagnosed without AAC. Participants who were older, non-Hispanic white, overweight, single, had moderate income levels, lower educational levels, smoked, and had conditions such as hypertension and diabetes exhibited higher prevalence rates of AAC.

### Analysis of AAC, MVPA, and sedentary behavior

Individuals who meet the U.S. Physical Activity Guidelines show a lower prevalence of AAC compared to those who do not, as demonstrated in S2 Table (25.97% vs. 35.71%, P < 0.001). Similarly, those who meet the physical activity guidelines in the occupation and leisure domains exhibit a lower prevalence of AAC compared to those who do not (occupation: 26.40% vs. 31.79%, P = 0.006; leisure: 23.86% vs. 32.95%, P < 0.001). However, the prevalence of AAC did not significantly differ between individuals who adhered to the guidelines for transport-related MVPA and those who did not (26.76% vs. 30.80%, P = 0.137). Additionally, significant differences in AAC prevalence were found among the Q1-Q4 groups categorized by sedentary time (P = 0.006). Post-hoc comparisons reveal that, compared to the Q1 group (<4 hours/day), both the Q3 group (≥6 to <8 hours/day) and the Q4 group (≥8 hours/day) have significantly higher AAC prevalence (24.83% vs. 32.73%, P = 0.034; 24.83% vs. 32.41%, P = 0.018).

Findings show that meeting the U.S. Physical Activity Guidelines is associated with a lower prevalence of AAC. As presented in S2 Table, individuals who met the overall guidelines had significantly lower AAC prevalence (25.97%) compared to those who did not (35.71%, P < 0.001). Lower AAC prevalence was also observed in specific activity domains for those meeting guidelines: in occupational MVPA (26.40% vs. 31.79% for those not meeting guidelines, P = 0.006) and leisure time MVPA (23.86% vs. 32.95%, P < 0.001). However, there was no significant difference in AAC prevalence based on meeting guidelines for transportation MVPA (26.76% vs. 30.80%, P = 0.137). In addition to physical activity, sedentary behavior time influenced AAC prevalence, showing significant differences across quartiles (P = 0.006). Post-hoc analysis revealed that the two groups with the most sedentary behavior time (Q3: ≥ 6 to <8 hours/day and Q4: ≥ 8 hours/day) had significantly higher AAC prevalence compared to the least sedentary group (Q1: < 4 hours/day). Specifically, Q3 prevalence was 32.73% (compared to 24.83% in Q1, P = 0.034), and Q4 prevalence was 32.41% (compared to 24.83% in Q1, P = 0.018).

### Multivariable logistic regression analysis of the impact of MVPA and sedentary behavior on AAC risk

The multivariable logistic regression analysis results on the relationship between MVPA, sedentary behavior, and AAC risk are presented in Table 1. Meeting leisure time MVPA guidelines was consistently associated with a significantly reduced risk of AAC across all three adjusted models compared to not meeting them. The OR for meeting versus not meeting guidelines were significant in Model 1 (OR=0.59, 95% CI 0.45–0.77, P = 0.002), Model 2 (OR=0.62, 95% CI 0.48–0.81, P = 0.011), and the fully adjusted Model 3 (OR=0.64, 95% CI 0.49–0.85, P = 0.035).

**Table 1:** Multivariable OR for AAC based on the Meeting Physical Activity Guideline (≥150 min/week).

	Model 1		Model 2		Model 3	
	OR (95%Cl)	P Value	OR (95% Cl)	P Value	OR (95% Cl)	P Value
Total MVPA (≥150 min/ week)						
No	1		1		1	
Yes	0.72	0.021	0.71	0.030	0.75	0.096
	(0.56-0.92)		(0.56-0.91)		(0.58-0.97)	
Occupational MVPA (≥150 min/ week)						
No	1		1		1	
Yes	1.03	0.815	0.99	0.953	1.01	0.923
	(0.82-1.30)		(0.78-1.26)		(0.80-1.29)	
Transportation MVPA (≥150 min/ week)						
No	1		1		1	
Yes	0.83	0.310	0.78	0.202	0.80	0.306
	(0.58-1.18)		(0.54-1.11)		(0.55-1.16)	
Leisure Time MVPA (≥150 min/week)						
No	1		1		1	
Yes	0.59	0.002	0.62	0.011	0.64	0.035
	(0.45-0.77)		(0.48-0.81)		(0.49-0.85)	
Sedentary Behavior (hours per day)						
Q1(<4)	1		1		1	
Q2(≥4 to <6)	0.86	0.393	0.87	0.464	0.89	0.608
	(0.61-1.20)		(0.62-1.23)		(0.61-1.30)	
Q3(≥6 to <8)	0.92	0.684	0.96	0.849	0.98	0.919
	(0.63-1.34)		(0.66-1.40)		(0.68-1.41)	
Q4(≥8)	1.12	0.426	1.23	0.181	1.22	0.262
	(0.86-1.46)		(0.95-1.60)		(0.95-1.56)	

Model 1 was adjusted for age and sex.

Model 2 is model 1, which is additionally adjusted for race, BMI, marital status, education level, and family income-poverty ratio.

Model 3 is model 2, which is additionally adjusted for smoking status, diabetes, and hypertension.

Abbreviations: CI, confidence interval; OR, odds ratio; PIR, poverty income ratio;

Abbreviations: AAC abdominal aortic calcification; MVPA moderate-to-vigorous physical activity.

Total MVPA also showed a reduced risk of AAC in the less-adjusted models. Specifically, the association was significant in Model 1 (OR=0.72, 95% CI 0.56–0.92, P = 0.021) and Model 2 (OR=0.71, 95% CI 0.56–0.91, P = 0.030). However, this relationship was no longer statistically significant after full adjustment for confounding factors in Model 3. Other types of MVPA, specifically occupation-related and transport-related, did not show a significant association with AAC risk in any of the tested models. Regarding sedentary behavior, in the fully adjusted Model 3, individuals in the highest quartile of sedentary time (Q4: ≥ 8 hours/day) showed a non-significant trend towards increased AAC risk compared to those in the lowest quartile (Q1: < 4 hours/day) (OR=1.22, 95% CI 0.95–1.56, P = 0.262).

### RCS analysis of MVPA, sedentary behavior, and AAC risk

**Fig 2:**
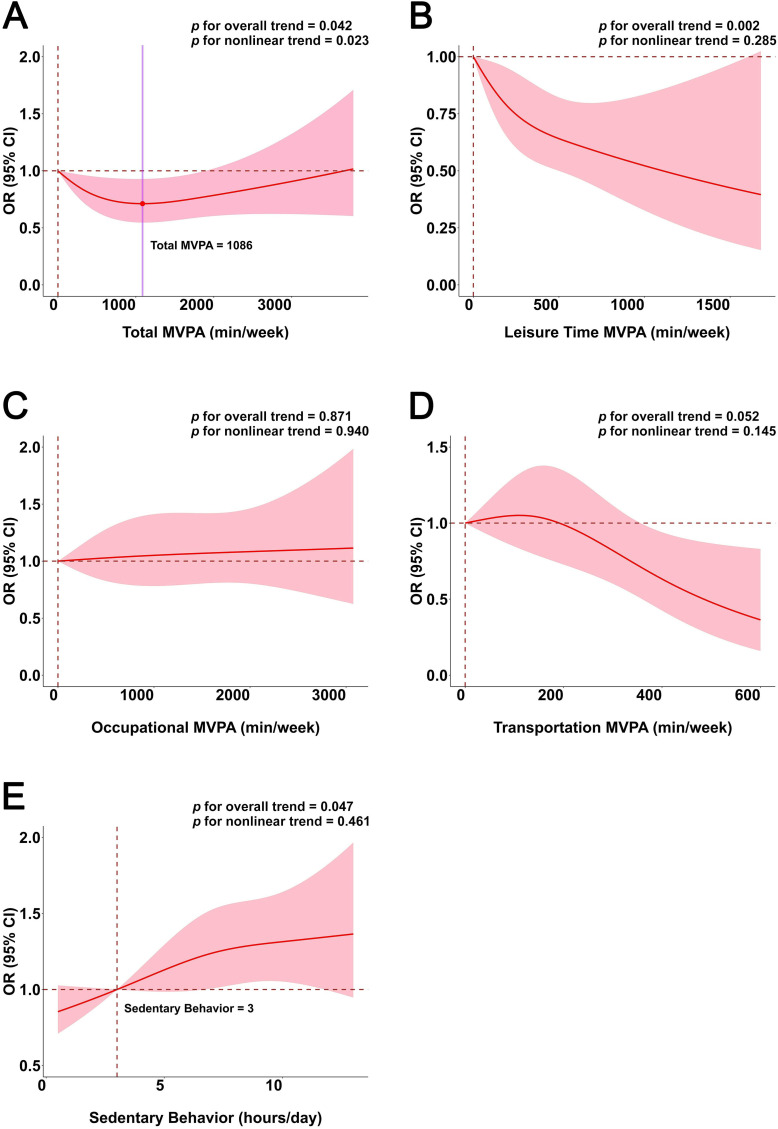
Dose–Response association between different domains of MVPA and sedentary behavior and the risk of AAC. Legends: **A** Total MVPA and AAC risk; **B** Leisure Time MVPA and AAC risk; **C** Occupational MVPA and AAC risk; **D** Transportation MVPA and AAC risk; **E** Sedentary behavior and AAC risk.

The relationship between total MVPA and AAC risk is approximately U-shaped (Fig 2A, P for overall = 0.042, P for nonlinear = 0.023). AAC risk declines with rising total MVPA, reaching its lowest point at approximately 1086 minutes (OR=0.712, 95% CI: 0.546–0.928), and gradually climbs. The relationship curve between leisure MVPA and AAC risk is approximately L-shaped (Fig 2B, P for overall = 0.002, P for nonlinear = 0.285), with AAC risk decreasing as duration extends. The relationship curve between sedentary behavior and AAC risk is J-shaped (Fig 2E, P for overall = 0.047, P for nonlinear = 0.461). When sedentary time is less than 3 hours/day, the curve indicates a negative OR; when it exceeds 3 hours/day, the curve demonstrates a positive OR and gradually rises. Occupation and transportation MVPA were not significantly associated with AAC risk (Fig 2C, P for overall = 0.871, P for nonlinear = 0.940; Fig 2DD, P for overall = 0.052, P for nonlinear = 0.145).

### Replacement effects of SB with PA

Table 2 presents results from isotemporal substitution models examining the associations of replacing 30 minutes per day of different activity types with AAC risk.Specifically, substituting 30 minutes/day of Leisure Time MVPA for SB (OR: 0.837, 95% CI: 0.747, 0.927) or Occupational MVPA (OR: 0.842, 95% CI: 0.692, 0.992) was significantly associated with lower AAC risk. Conversely, substituting 30 minutes/day of SB (OR: 1.163, 95% CI: 1.013, 1.313) or Occupational MVPA (OR: 1.158, 95% CI: 1.008, 1.308) for Leisure Time MVPA was significantly associated with higher AAC risk. Substitutions involving Transportation MVPA or mutual substitutions between SB and Occupational MVPA for 30 minutes per day did not show statistically significant associations with AAC risk.

**Table 2:** Isotemporal substitution models of associations of 30 min/day of sedentary behaviors, and physical activity with AAC risk.

	(30 min/day)	SB OR (95% CI)	Occupational MVPA OR (95% CI)	Transportation MVPA OR (95% CI)	Leisure Time MVPA OR (95% CI)
Isotemporal substitution model (ISM)*	Substitute SB (a)	Replaced	0.984 (0.949,1.019)	0.863 (0.689,1.037)	**0.837** **(0.747,0.927)**
	Substitute Occupational (b)	1.016 (0.981,1.054)	Replaced	0.878 (0.698,1.057)	**0.842** **(0.692,0.992)**
	Substitute Transportation (c)	1.137 (0.987,1.287)	1.122 (0.972,1.272)	Replaced	0.983 (0.850, 1.120)
	Substitute Leisure Time (d)	**1.163** **(1.013,1.313)**	**1.158** **(1.008,1.308)**	1.017 (0.817, 1.217)	Replaced

SB: sedentary behavior; MVPA: moderate to vigorous physical activity; CI: confidence interval; AAC abdominal aortic calcification.

* ISM was adjusted for sex, age, race, marital status, education level, family income-poverty ratio, BMI, smoking status, diabetes, and hypertension, and total behavior time, the type of activity other than the dropped one (if sedentary time was dropped, then occupational MVPA, leisure time MVPA and transportation MVPA were adjusted for; if occupational MVPA was dropped, then leisure time MVPA, transportation MVPA and sedentary time were adjusted for; if transportation MVPA was dropped, then occupational MVPA, leisure time MVPA, and sedentary time were adjusted for; if leisure time MVPA was dropped, then occupational MVPA, transportation MVPA, and sedentary time were adjusted for).

(a) SB was replaced by physical activities.

(b) occupational MVPA was replaced by SB, transportation MVPA and leisure time MVPA.

(c) transportation MVPA was replaced by SB, occupational MVPA and leisure time MVPA.

(d) leisure time MVPA was replaced by SB, occupational MVPA and transportation MVPA.

## Discussion

To our knowledge, this study is the first to analyze the dose-response and isotemporal substitution relationships between MVPA in different domains, sedentary behavior, and the risk of AAC. The results showed that individuals who adhered to the leisure-time MVPA guidelines were observed to have a significantly lower AAC risk compared to those who did not follow the guidelines. Importantly, this study revealed distinct dose-response associations for different physical activity domains and sedentary behavior with AAC risk: total MVPA exhibited a nonlinear, approximately U-shaped association with AAC risk, showing the lowest risk observed at approximately 1086 minutes per week; leisure-time MVPA displayed an L-shaped association with AAC risk; and sedentary behavior showed a J-shaped association. Additionally, isotemporal substitution analysis revealed that replacing 30 minutes of work MVPA or sedentary time with leisure-time MVPA was associated with a lower risk of AAC.

Existing research investigating the relationship between physical activity, sedentary behavior, and vascular calcification is relatively limited and has primarily focused on CAC or the progression of AAC scores. While a study by Sheng et al. [23] examined the association between physical activity, sedentary behavior, and the progression of AAC, there remains a lack of studies that systematically assess the association between these behaviors and the risk of developing AAC itself. While some prospective cohort studies have reported associations between regular physical activity and lower CAC risk(e.g., MESA [38], SCAPIS [39], JHS [40]), and others have explored the relationship between physical activity and AAC progression [23–25], detailed analyses of the dose-response patterns and isotemporal substitution effects of different physical activity domains and sedentary behavior on AAC risk are largely absent in the existing literature.

Sheng et al. [23] previously analyzed the relationship between physical activity and sedentary behavior and AAC scores, reporting results that suggested participation in occupational MVPA and reducing sedentary behavior might help lower AAC scores. However, the results of the current study differ significantly from Sheng et al.‘s analysis in several key aspects, particularly regarding the impact of different types of physical activity on AAC risk. In contrast to the emphasis on the potential benefits of occupational MVPA in Sheng et al.’s analysis, our dose-response analysis found no significant association between occupational MVPA and lower AAC risk. Instead, we observed a significant association with leisure time MVPA. This divergence suggests that the cardiovascular protective effects of physical activity may vary significantly depending on the context in which the activity occurs. Specifically, our findings indicate that substituting leisure time MVPA for occupational MVPA or sedentary behavior is associated with a lower AAC risk. This observation aligns with the concept of the ‘physical activity paradox’ [27], which posits that not all forms of physical activity confer equivalent cardiovascular benefits.Several factors may contribute to the differences between our study and that of Sheng et al. While they reported benefits of occupational MVPA for the progression of AAC, we did not find a significant association with reduced AAC risk. One possible explanation is the differing contexts of occupational and leisure time MVPA. Occupational activities are often perceived as obligatory and may involve higher stress levels, potentially diminishing their cardiovascular benefits. In contrast, leisure activities are typically associated with enjoyment and lower stress, which may enhance their health benefits. Additionally, socioeconomic and psychosocial factors surrounding occupational MVPA—such as low job control, high job demands, and limited social support—may further attenuate its health benefits. These contextual differences may help explain the divergence in findings between our study and that of Sheng et al., despite both utilizing the same data.

Consistent with the findings of this study, a cohort study [26] from Copenhagen, which included 104,046 adults with a median follow-up period of 10 years, reported that increased leisure-time physical activity is associated with a reduced risk of major adverse cardiovascular events (MACE). This study further identified an observed dose-response relationship association between leisure-time MVPA and the risk of AAC. Additionally, it was observed that MVPA associated with transportation or occupation activities did not have a significant relationship with lower AAC risk. In alignment with our findings, extensive research has shown that physical activities related to transportation or occupation do not provide the same health benefits as leisure-time physical activities. Treff et al. [41] indicated that individuals more actively engaged in transportation activities might be more susceptible to the adverse effects of transportation-related air pollution. Similarly, Cutrufello et al. [42] found that exercising in controlled environments with exposure to air pollution can alter vascular injury markers, and increase arterial stiffness, and vascular reactivity, thereby diminishing exercise capacity. This evidence suggests that transportation-related air pollution may significantly reduce the cardiovascular benefits typically associated with transportation activities. A Danish cohort study [43] found that work-related physical exposure is linked to long-term sick leave and negatively impacts physical health. Moreover, the characteristics of occupational physical activity can vary widely, often involving repetitive motions, heavy lifting, or sustained strenuous effort, which under certain conditions (e.g., unfavorable environmental exposure or specific job types) can contribute to increased inflammation [44,45] and induces oxidative stress [46]. Both inflammation and oxidative stress are critical drivers of vascular calcification [47,48]. Another important finding of this study is that prolonged sedentary behavior correlates with a higher risk of AAC, aligning with existing literature on the heightened risk of cardiovascular diseases and coronary artery calcification because of sedentary behavior [21,49,50]. Additionally, this study identified a dose-response relationship between sedentary behavior and the risk of AAC. While existing studies have primarily focused on the association between sedentary behavior and AAC progression [23] with findings often indicating that increased sedentary time is associated with faster progression, few studies have explored the direct association between sedentary behavior and the risk of AAC onset. The results of the current study indicate a significant association between sedentary behavior and the risk of AAC onset. Therefore, these findings build upon and extend previous work, suggesting that sedentary behavior may not only accelerate the deterioration of AAC but could also be a significant factor in its initial occurrence. This further highlights the critical importance of limiting sedentary time for AAC prevention.

The results of this study indicate a nonlinear dose-response association between total MVPA levels and AAC risk, characterized by an approximately U-shaped curve. It was observed that as MVPA levels increase, AAC risk initially appeared to decrease and then gradually increase. This is consistent with previous research suggesting that the relationship between physical activity and atherosclerosis is level-dependent. A negative correlation has been shown at low levels of physical activity [51], while some studies investigating very high volumes of exercise have reported a plateauing of benefits or even less favorable cardiovascular outcomes at supra-maximal levels [52]. Further context is provided by investigations into the effects of very high volumes of exercise on cardiovascular outcomes. For instance, a ten-year longitudinal study [53] reported an 11% higher risk of coronary artery disease in men engaging in more than 3,000 MET-min/week compared to those with low-intensity exercise. This observation, particularly when contrasted with our finding of reduced AAC risk at high MVPA levels (up to 1086 minutes/week), suggests that while significant benefits exist across a wide range of activity levels, the dose-response relationship for vascular outcomes may become less favorable or even plateau at potentially supra-maximal volumes. The observed variability in outcomes at high and very high activity levels across different studies may be attributable to a range of factors. These include potential confounders such as underlying preclinical cardiovascular disease, genetic factors influencing exercise response or disease susceptibility, and lifestyle variables. Furthermore, methodological differences in physical activity assessment, variation in exercise modalities or training regimens, insufficient recovery periods, and heterogeneity in defined cardiovascular endpoints (e.g., AAC vs. CAD) could contribute to disparate findings.

This study’s strengths include a large, nationally representative sample of U.S. adults and the use of validated physical activity and sedentary behavior questionnaires administered by trained personnel following a systematic protocol. These measures ensured the findings’ generalizability, accuracy, and reliability at a national population level. Additionally, the study examined the dose-response relationship between physical activity in various domains and sedentary behavior with AAC risk, identifying a potential nonlinear relationship between MVPA and AAC risk. This fills a gap in the evidence and provides reference information for future physical activity and sedentary behavior guidelines.

This study has limitations. Sedentary time and physical activity were self-reported, not device-measured. Although wearable devices often measure physical activity and monitor sedentary time, most, except thigh-mounted monitors, cannot differentiate between prolonged sitting and sedentary behaviors (e.g., lying down or reclining) and may misclassify stationary behaviors (e.g., standing) as sedentary time. Moreover, device measurement for just one week may not accurately reflect habitual physical activity levels. As a cross-sectional study, it is limited by its observational design and cannot establish causality. Longitudinal research is necessary to elucidate the cause-and-effect relationship between physical activity, sedentary behavior, and the risk of AAC. Finally, residual confounding by unmeasured factors, such as dietary intake, medication use, or other co-occurring diagnostic conditions, cannot be entirely ruled out despite multivariable adjustment.

## Conclusion

There is a positive linear dose-response relationship between sedentary behavior and AAC risk. Conversely, leisure-time MVPA demonstrates a negative linear dose-response relationship with AAC risk. Furthermore, total MVPA presents a nonlinear dose-response relationship, with AAC risk being lowest when activity reaches 1086 minutes per week. Moreover, reallocating time from sedentary behavior or occupational MVPA to leisure-time MVPA was significantly associated with lower AAC risk. These findings collectively suggest that appropriately increasing physical activity, especially by prioritizing leisure-time activities, and reducing sedentary behavior may help optimize AAC risk.

## Supporting information

S1 Table The baseline characteristics of participants. (PDF)

S2 Table AAC status by meeting physical activity guidelines and sedentary behavior levels. (PDF)
