# Supplementary material for: Dose-response and isotemporal substitution analysis of domain-specific physical activity and sedentary behavior with abdominal aortic calcification risk: A cross-sectional study
Source: PLoS One. 2025 Oct 7;20(10):e0332964. doi: 10.1371/journal.pone.0332964 (PMC12503245; doi:10.1371/journal.pone.0332964)
Supplement: S1 Table — (PDF) [file pone.0332964.s001.pdf]

S1 Table. The baseline characteristics of participants.

Characteristic	All (n =2842) (%)	Non-AAC (n =1981) (% of All)	AAC (n =861) (% of All)	P Value Yes/No
Age, years, (median [IQR])	58 [48-68]	55 [46-64]	66 [55-75]	<0.001
Sex, n (%)				0.342
Male	1376 (48.42%)	947 (47.80%)	429 (49.83%)	
Female	1466 (51.58%)	1034 (52.20%)	432 (50.17%)	
Race, n (%)				<0.001
Mexican American	348 (12.24%)	265 (76.15%)	83 (23.85%)	
Other Hispanic	255 (8.97%)	190 (74.51%)	65 (25.49%)	
Non-Hispanic White	1284 (45.18%)	811 (63.16%)	473 (36.84%)	
Non-Hispanic Black	567 (19.95%)	425 (74.96%)	142 (25.04%)	
Non-Hispanic Asian	331 (11.65%)	249 (75.23%)	82 (24.77%)	
Other Race	57 (2.01%)	41 (71.93%)	16 (28.07%)	
BMI, n (%)				<0.001
Normal (< 25 kg/m^2)	806 (28.36%)	548 (67.99%)	258 (32.01%)	

Overweight (≥ 25 and < 30 kg/m^2)	1032 (36.31%)	677 (65.60%)	355 (34.40%)	
Obese (≥ 30 kg/m^2)	1004 (35.33%)	756 (75.30%)	248 (24.70%)	
Marital status, n (%)				<0.001
Married/Living with partner	1798 (63.27%)	1304 (72.53%)	494 (27.47%)	
Single	1044 (36.73%)	677 (64.85%)	367 (35.15%)	
Education level, n (%)				<0.05
High school degree/equivalency or less	1259 (44.30%)	852 (67.67%)	407 (32.33%)	
Some college or associates degree	821 (28.89%)	571 (69.55%)	250 (30.45%)	
College Graduate or above	762 (26.81%)	558 (73.23%)	204 (26.77%)	
Family income-poverty ratio, n (%)				<0.01
<1.38	887 (31.21%)	610 (68.77%)	277 (31.23%)	
≥1.38 and <3.99	1128 (39.69%)	756 (67.02%)	372 (32.98%)	
≥3.99	827 (29.10%)	615 (74.37%)	212 (25.63%)	
Smoking status, n (%)				<0.001
Never	1501 (52.81%)	1111 (74.02%)	390 (25.98%)	
Former	539 (18.97%)	358 (66.42%)	181 (33.58%)	
Now	802 (28.22%)	512 (63.84%)	290 (36.16%)	

Diabetes, n (%)				<0.001
Yes	667 (23.47%)	339 (59.82%)	268 (40.18%)	
No	2175 (76.53%)	1582 (72.74%)	593 (27.26%)	
Hypertension, n (%)				<0.001
Yes	1530 (53.84%)	947 (61.90%)	583 (38.10%)	
No	1312 (46.16%)	1034 (78.81%)	278 (21.19%)	

Numbers are IQR interquartile range or n (%).

Abbreviations: BMI, body mass index; PIR poverty income ratio;

AAC: abdominal aortic calcification
