# Supplementary material for: Dose-response and isotemporal substitution analysis of domain-specific physical activity and sedentary behavior with abdominal aortic calcification risk: A cross-sectional study
Source: PLoS One. 2025 Oct 7;20(10):e0332964. doi: 10.1371/journal.pone.0332964 (PMC12503245; doi:10.1371/journal.pone.0332964)
Supplement: S2 Table — (PDF) [file pone.0332964.s002.pdf]

S2 Table. AAC status by meeting physical activity guidelines and sedentary behavior levels.

Characteristic	All (n=2842) (%)	AAC		
		No (n=1981) (% of All)	Yes (n=861) (% of All)	P Value
Total MVPA (≥150 min/ week)				< 0.001
No	1263(44.44)	812(64.29)	451(35.71)	
Yes	1579(55.56)	1169(74.03)	410(25.97)	
Occupational MVPA (≥150 min/ week)				0.006
No	2054(72.27)	1401(68.21)	653(31.79)	
Yes	788(27.73)	580(73.60)	208(26.40)	
Transportation MVPA (≥150 min/ week)				0.137
No	2487(87.51)	1721(69.20)	766(30.80)	
Yes	355(12.49)	260(73.24)	95(26.76)	
Leisure Time MVPA (≥150 min/ week)				< 0.001
No	2012(70.80)	1349(67.05)	663(32.95)	
Yes	830(29.20)	632(76.14)	198(23.86)	

Sedentary Behavior (hours per day)				0.006
Q1(<4)	451(15.87)	339(75.17)	112(24.83)	
Q2(≥4 to <6)	578(20.34)	418(72.32)	160(27.68)	
Q3(≥6 to <8)[a]	440(15.48)	296(67.27)	144(32.73)	
Q4(≥8)[b]	1373(48.31)	928(67.59)	445(32.41)	

The study defined meeting physical activity guidelines as an individual meeting the guidelines (150 minutes/week of moderate-intensity activity and 75 minutes/week of vigorous-intensity activity for adults, or equivalent) based on the 2018 Physical Activity Guidelines for Americans. combination) based on various physical activities.

Abbreviations: AAC abdominal aortic calcification; MVPA moderate-to-vigorous physical activity.

[a] Indicates P = 0.034 compared with Q1 groups;

[b] Indicates P = 0.018 compared with Q1 groups.
